# Aidi Injection, Compound Kushen Injection, or Kanglaite Injection: Which Is the Best Partner with Systemic Chemotherapy for Patients with HCC? A Network Meta-Analysis

**DOI:** 10.1155/2020/5497041

**Published:** 2020-08-21

**Authors:** Dou Dou, Ze-yu Zhang, Zhi-yuan Wu, Xu-dong Qiu, Xiang-gen Zhong

**Affiliations:** ^1^School of Chinese Medicine, Beijing University of Chinese Medicine, Beijing, China; ^2^China-Japan Friendship Hospital, Beijing, China; ^3^School of Public Health, Capital Medical University, Beijing, China

## Abstract

**Objective:**

The aim of this network meta-analysis (NMA) was to explore the effectiveness of different traditional Chinese medicine injections (TCMIs) combined with systemic chemotherapy for the treatment of hepatocellular carcinoma (HCC).

**Methods:**

A comprehensive search for randomized controlled trials (RCTs) was performed with regard to different TCMIs for treating HCC in seven electronic databases up to November 2019. The quality assessment of the included RCTs was conducted according to the Cochrane risk of bias tool. The objective response rate (ORR), clinical benefit rate (CBR), and Karnofsky performance score (KPS) data were extracted. The network meta-analysis used the network package in Stata software to analyse the data and draw a map of the evidence summarizing the direct and indirect comparisons.

**Results:**

A total of 1697 articles were retrieved through the comprehensive search. Twenty RCTs focusing on Aidi injection, compound Kushen injection, and Kanglaite injection as adjuvant therapies to chemotherapy were included, involving a total of 1418 patients. The NMA statistics showed that all three indicators (ORR, CBR, and KPS) were better in the combined treatment group of TCMIs with chemotherapy than that in the single treatment group of chemotherapy alone. Kanglaite injection tended to be better than the other two in terms of primary outcome, but there was not a significant difference. The combined treatment group had fewer adverse reactions than the single treatment group. Moreover, several articles reported that TCMIs combined with chemotherapy could increase the number of CD3+ and CD4+ T lymphocytes and the ratio of CD4+/CD8+ T lymphocytes.

**Conclusions:**

TCMIs combined with systemic chemotherapy could be an effective and safe treatment option for patients with HCC. Kanglaite injection showed a tendency to be better than the other two kinds of injections in terms of ORR. Nevertheless, additional results from multicentre trials and high-quality studies will be pivotal for supporting our findings.

## 1. Introduction

Hepatocellular carcinoma (HCC) is one of the most common malignant tumours. Its incidence has increased significantly in recent years. The latest statistics show that there are approximately 840,000 new cases of HCC each year worldwide. In addition, HCC is the third leading cause of cancer-related death. In 2018 alone, 780,000 patients worldwide died of HCC [1].

The onset of HCC is subtle, and there is no practical method for diagnosing HCC at the early stage. Although diagnostic methods and public awareness of the disease have improved in recent years, most patients have reached the late stage when they are diagnosed; thus, surgery is not an option for these patients, and their prognosis is poor [2, 3].

Although chemotherapy has limited efficacy in advanced HCC, it is still a treatment option in patients who cannot receive transcatheter arterial chemoembolization (TACE) treatment [3–5]. Improving the efficacy of systemic chemotherapy and reducing its adverse reactions have become major concerns for researchers.

China has a high incidence of HCC. The number of new cases and deaths each year is 394,770 and 383,203, respectively, accounting for half of the global number [6]. Therefore, a large number of Chinese research teams have invested in HCC research, and many of them concentrate on Chinese medicine. Recent studies have indicated that traditional Chinese medicine (TCM) could play an essential role in the whole course of HCC treatment [7].

Traditional Chinese medicine injection (TCMI) is a convenient and effective new method of administering Chinese medicine. The latest Chinese Society of Clinical Oncology (CSCO) guidelines for the diagnosis and treatment of HCC recommend a variety of TCMIs for the treatment of late-stage HCC [8], and there have been numerous related randomized controlled trials (RCTs). The majority of the studies focused on TCMIs combined with TACE, while studies on TCMIs combined with systemic chemotherapy were limited, and there was no systematic summary on the latter. Moreover, no comparison has been made between different TCMIs used in combination with chemotherapy.

One approach to create a systematic summary is a network meta-analysis, which is a technique that combines direct evidence and indirect evidence to compare multiple treatment options. This study aims to make various comparisons between a range of TCMIs combined with systemic chemotherapy and systemic chemotherapy alone in the treatment of patients with HCC by using data from available RCTs in a network meta-analysis.

## 2. Materials and Methods

### 2.1. Inclusion and Exclusion Criteria

#### 2.1.1. Inclusion Criteria

The inclusion criteria include the following: ① research subjects: patients with HCC whose diagnoses were confirmed by imaging (B-ultrasound, CT, or MRI) and alpha-fetoprotein examination or by pathological biopsy; ② intervention measures: TCMIs combined with systemic chemotherapy (intravenous or oral chemotherapy drugs) compared with systemic chemotherapy alone; ③ study type: randomized controlled trial; ④ end point event: objective response rate (abbreviated as ORR, calculated by (CR + PR)/total number) and clinical benefit rate (abbreviated as CBR, calculated by (CR + PR + SD)/total number), both evaluated by RECIST criteria.

#### 2.1.2. Exclusion Criteria

The inclusion criteria include the following: ① studies without a precise chemotherapy regimen; ② studies with apparent data errors; and ③ studies without sufficient available data.

### 2.2. Literature Source and Retrieval

A combination of MeSH words and free words was used to develop a search strategy based on the Cochrane Handbook for Systematic Review of Interventions (Version 5.1.0) [9]. We conducted a systematic publication review of seven databases, including MEDLINE, EMBASE, Cochrane Library, Chinese National Knowledge Infrastructure (CNKI), China Biology Medicine (CBM), VIP database, and Wanfang database. All documents were retrieved by November 11, 2019.

### 2.3. Literature Screening, Data Extraction, and Quality Evaluation

Two researchers independently read the context of the literature, excluded studies that did not meet the inclusion criteria, and cross-checked the results of the included trials. Disagreement was resolved through discussions or a third investigator. The extracted data mainly included the basic information included in the study, the characteristics of the study object, intervention measures, outcomes, and other information. The two researchers independently extracted data according to a predesigned form and evaluated the quality. Repeated reports were combined into one. The bias risk and the quality of RCTs were evaluated according to the “bias risk assessment” tool recommended by Cochrane Handbook 5.1.

### 2.4. Statistical Analysis

The network meta-analysis used the network package in Stata to analyse data and draw a map of the evidence to summarize the direct and indirect comparisons. If the consistency test showed no inconsistency in the comparison of curative effects among intervention methods, the results were analysed by the consistency model. Otherwise, an inconsistency model was used. The maximum likelihood method was used to select the most stable model in multiple comparisons. After 5000 iterations, the final order of the recommended intervention measures was obtained, the pairwise comparison results of the four intervention measures were summarized, and the OR value was used to show the curative effect of different intervention measures. Finally, a funnel chart was used to show publication bias. If most of the studies were located between the dotted lines, publication bias could be ignored.

## 3. Results

### 3.1. Literature Search and Screening Results

A total of 1697 articles were retrieved, 198 duplicates were excluded, and 1499 were left. Among them, 1421 articles were excluded by reading the title and abstract. The reasons for exclusion were animal or cell experiments, the research object was not HCC, the use of oral or topical Chinese medicine instead of TCMIs, or the treatment options were arterial interventional chemotherapy or intraperitoneal infusion chemotherapy instead of systemic chemotherapy.

Seventy-eight articles were initially selected, and six articles were manually retrieved according to the references of the selected papers. After reading 84 full texts, 64 articles were further excluded. The reasons for exclusion were basic experiments or reviews (6 articles), clinical studies but not RCTs (10 articles), multicarcinoma studies including HCC patients but no subgroup data of HCC explicitly reported (3 articles), not combined with systemic chemotherapy (25 articles), no reporting of the specific chemotherapy regimen (4 articles), dosage form of the traditional Chinese medicine was not an injection (3 articles), ORR and CBR were not clearly reported (6 articles), apparent data errors (1 article), and the number of articles about a particular Chinese medicine injection was only one (6 articles).

**Figure 1:**
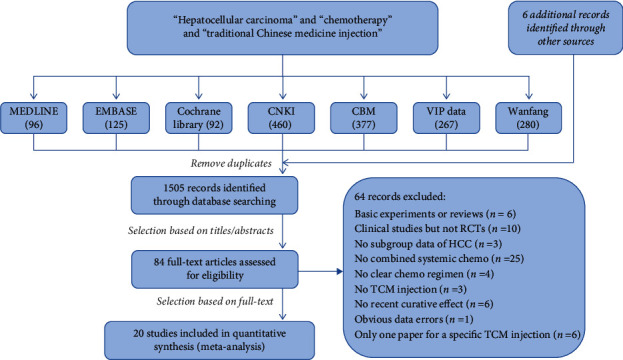
Literature screening flowchart.

**Figure 2:**
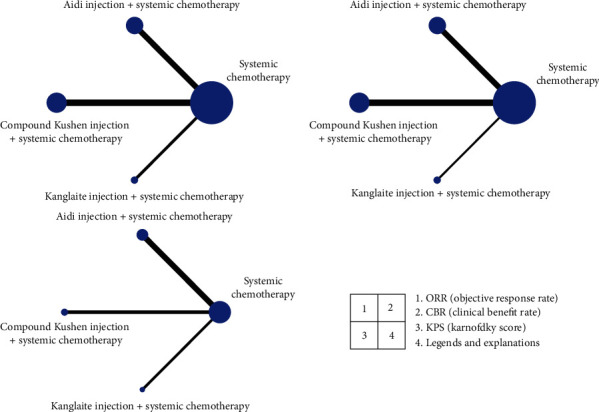
Network meta-analysis of available comparisons. Line width is proportional to the number of trials including every pair of treatments. Circle size is proportional to the total number of patients for each treatment in the network.

Twenty RCT studies [10–29] were included. Eight of them were about Aidi injection, nine about compound Kushen injection, and three about Kanglaite injection. The literature screening flowchart is shown in Figure 1, and the network meta-analysis of available comparisons is shown in Figure 2.

### 3.2. Characteristics and Quality Evaluation of the Included Studies

The twenty included studies were published from August 15, 2002, to November 8, 2018, involving a total of 1,418 patients. The number of patients included in the studies varied from 30 to 150. All of the studies reported the patients' sex, age range, and mean age. Each study claimed that there was no significant difference in sex and age between the combined treatment group and the single treatment group. Sixteen studies reported TNM staging of tumours, one of which enrolled patients without stage limitation, seven of which enrolled patients with stage II and above, and eight of which enrolled patients with stage III and above. Two studies reported the patients' Child–Pugh score, two reported subtypes of HCC, and five reported the Karnofsky performance score (KPS) at enrolment.

Interventions in the twenty studies were TCMIs combined with systemic chemotherapy, while the single treatment group only received systemic chemotherapy. TCMIs include Aidi injection, compound Kushen injection, and Kanglaite injection. The chemotherapy regimen included 13 combined chemotherapy regimens based on oxaliplatin or cisplatin, three combined chemotherapy regimens based on fluorine drugs, and four single-agent chemotherapy regimens. The dosages of TCMIs were the regular dosages of their respective drug instructions, which were Aidi injection 50–100 ml once a day, compound Kushen injection 10–30 ml once a day, and Kanglaite injection 200 ml once a day. Eighteen studies reported the course of treatment, nine of which had two cycles, eight of which had three to four cycles, and one of which had six cycles. The duration of the treatment ranged from 20 to 126 days, with an average of 61.2 days (for details of the characteristics of the studies, see Table 1).

**Figure 3:**
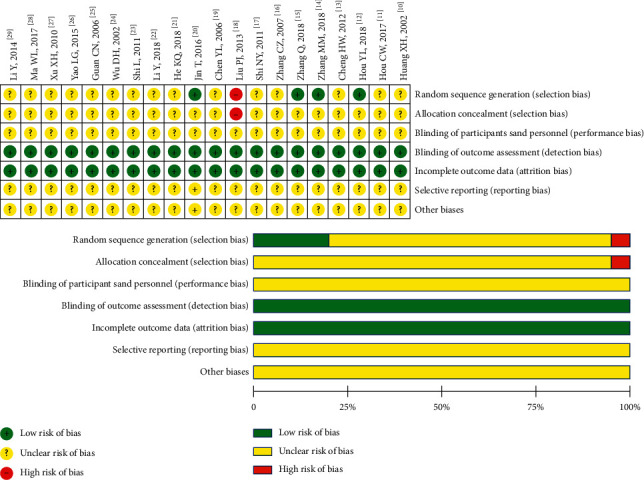
Assessment of risk of bias.

Of all the included studies, five studies reported detailed random allocation methods, and the others only mentioned randomness without description; two studies reported the number of patients lost to follow-up; no study mentioned whether the blind method was used or whether the allocation was hidden; all of the studies reported ORR as the primary outcome. The risk of bias and the quality of RCT studies included in this study were acceptable, as shown in Figure 3.

### 3.3. Network Meta-Analysis Results

**Figure 4:**
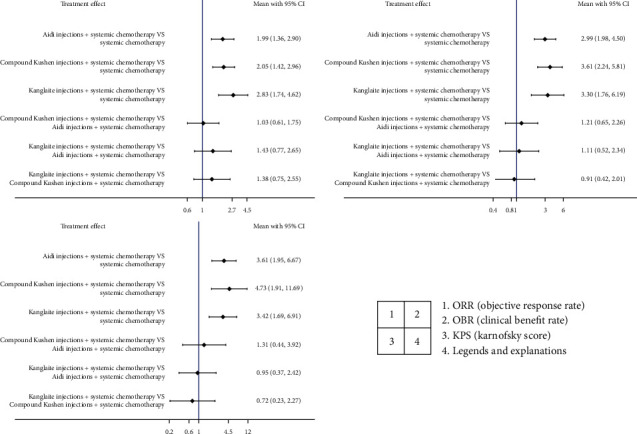
Forest plots (results of network meta-analysis of 3 kinds of TCMI in treatment of HCC).

**Figure 5:**
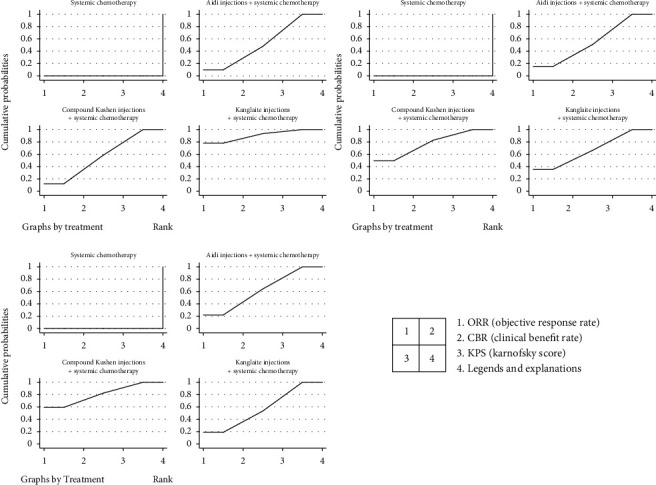
Surface under the cumulative ranking curve (SUCRA).

ORR was the primary outcome, and CBR and quality of life (evaluated by KPS, an improvement was considered when the patient's KPS rose by more than 10 points) were the secondary outcomes. We used fixed-effect models to analyse them. The statistics showed that all three indicators (ORR, CBR, and KPS) were better in the TCMI combined with the chemotherapy group than in the chemotherapy alone group. In terms of the differences among the three TCMIs, Kanglaite injection tended to be better than the other two in terms of the primary outcome, but it did not show a significant difference (Figures 4 and 5).

Based on the three indicators, the three different TCMIs were ranked: Kanglaite injections and compound Kushen injections were better than Aidi injections.

**Figure 6:**
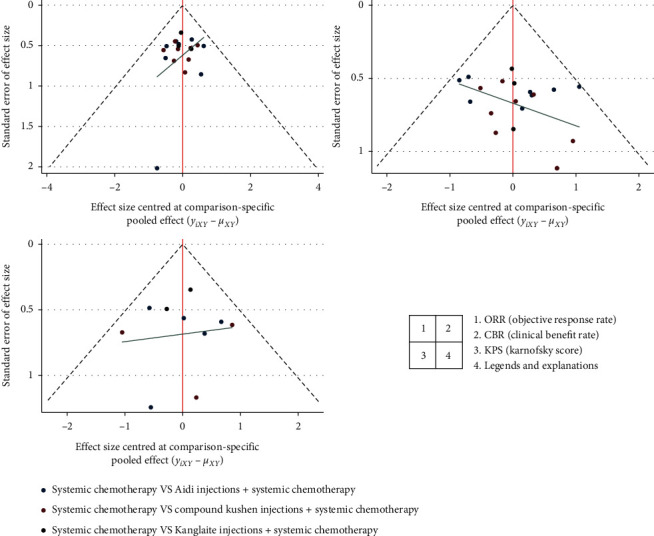
Funnel plots measuring the publication bias and small-sample effects of included RCTs.

Publication bias of the included RCTs was measured by funnel plots (Figure 6), which showed that the publication bias in this study can be ignored.

### 3.4. Reports of Other Efficacy Indicators

A total of 6 studies reported efficacy indicators other than ORR and CBR, such as time to progression (TTP), overall survival (OS), and median survival time (MST).

Three studies focused on Aidi injection. A study that used 5-FU chemotherapy showed that the 6-month OS in the combined treatment group and the single treatment group was 66% and 28.9%, respectively [10]. Moreover, the 1-year survival rates were 26.7% and 0%. Another study combining Aidi injection and FOLFOX4 [13] showed that the TTP of the combined treatment group and the single treatment group was 8.6 months and 6.5 months, respectively. There was also a study combining Aidi injection and cisplatin plus epirubicin [17]; the OS at six months in the combined treatment group and the single treatment group was 25.7% and 20%, respectively, and the OS at nine months was 20% and 14.3%.

There was only one study on compound Kushen injection. This study, which only included patients with stage III HCC [25], showed that the 1-year OS rates were 45% and 27.8% in the combined treatment group and single treatment group, respectively. Its chemotherapy regimen was a combination of fluorine and platinum.

There were two studies on Kanglaite injection. A study with capecitabine showed that [27] TTP was 5.7 ± 2.6 months and 4.4 ± 2.3 months in the combined treatment group and single treatment group, respectively, while the MST was 11.8 ± 6.4 months and 8.8 ± 5.7 months, respectively. Another study [29] of Kanglaite injection combined with doxorubicin chemotherapy showed that the 1-year OS was 88% and 68% in the two groups. This study included 18% early-stage patients.

### 3.5. The Influence on the Immune System

A total of four studies reported the effects of TCMIs on patients' immune systems. Among them, two studies focused on Aidi injection. One study involving 86 patients with HCC [11] showed that the number of CD3^+^ T lymphocytes and CD4^+^ T lymphocytes and the ratio of CD4^+^/CD8^+^ T lymphocytes in the combined treatment group increased after treatment, while the above indicators in the single treatment group decreased, and there was a significant difference between the two groups (*P* < 0.05). Another study involving 70 patients with HCC [17] showed that CD3^+^ T lymphocytes, CD4^+^ T lymphocytes, and CD8^+^ T lymphocytes in the combined treatment group increased after treatment. In contrast, the above indicators in the single treatment group decreased, with a significant difference between the two groups (*P* < 0.05), but the change in the ratio of CD4^+^/CD8^+^ T lymphocytes was not significantly different.

There were two studies on Kanglaite injection. One study involving 86 patients with HCC [28] showed that there was no significant difference in the levels of CD3^+^ T lymphocytes and CD4^+^ T lymphocytes or the ratio of CD4^+^/CD8^+^ T lymphocytes between the two groups before chemotherapy (*P* > 0.05), while all three indicators increased significantly in both groups after treatment compared with before (*P* < 0.05). Additionally, the above immune indexes were significantly higher in the combined treatment group than in the single treatment group (CD3^+^ T lymphocytes, CD4^+^ T lymphocytes, *P* < 0.01; CD4/CD8, *P* < 0.05). Another study involving 150 HCC patients [29] showed that CD3^+^ T lymphocytes, CD_4_^+^ T lymphocytes, and the ratio of CD4^+^/CD8^+^ T lymphocytes in the combined treatment group were significantly increased after treatment (*P* < 0.05), while the above indicators in the single treatment group were not significantly changed.

### 3.6. Reports of Adverse Effects

A total of 13 studies reported the safety and adverse effects of the treatments. During the treatments, adverse reactions were controllable, and no patient discontinued treatment because of adverse reactions. A total of 14 adverse reactions were reported: digestive system reactions, including nausea, vomiting, diarrhoea, abdominal pain, and abnormal liver function; bone marrow suppression, including leukopenia, anaemia, and platelet decline; skin and mucosal reactions, including erythema dermatitis, oral ulcers, and urticaria; and other adverse reactions, including fever, phlebitis, hair loss, and abnormal renal function.

Five articles on Aidi injection showed a significantly lower incidence of adverse reactions in the combined treatment group than in the single treatment group, and the *P* value reported in each study was less than 0.05 (except for the incidence of phlebitis).

The incidence of adverse reactions in 5 of 6 articles on compound Kushen injection was significantly lower in the combined treatment group than in the single treatment group. Another article [24] reported that the incidence of adverse reactions was low in both groups without a significant difference.

Among the two articles of Kanglaite injection, one showed that nausea, vomiting, and hair loss were significantly reduced after the combination of chemotherapy with Chinese medicine. However, there was no significant decrease in the incidence of bone marrow suppression and abdominal pain and diarrhoea. The other article did not show a significant difference in adverse reactions between the two groups.

## 4. Discussion

The rate of early diagnosis of HCC is low, and most patients have lost their chance of surgery at the time of diagnosis. Although there are a large number of interventional therapies for these patients, some patients cannot tolerate invasive procedures. An international multicentre RCT (each study) involving 371 patients with late-stage HCC who were no longer eligible for interventional therapy [30] found that FOLFOX4 systemic chemotherapy based on oxaliplatin could significantly improve patients' mPFS, ORR, DCR, and mOS. In summary, systemic chemotherapy is still an alternative for patients with advanced HCC.

In China, a country with a high incidence of HCC, researchers have developed many different types of TCMIs, which are injections extracted from animal or plant natural medicines that have been widely used as adjuvant treatments for advanced HCC and have been written into the CSCO guidelines [8]. Numerous RCTs, systematic reviews, and meta-analyses have illustrated the role of TCM in the management of hepatocellular carcinoma [31, 32]. A recent large study involving 3483 patients with HCC compared TCM users with non-TCM users and found that using TCM as adjuvant therapy can probably prolong median survival time and improve overall survival in patients with HCC [33].

Many kinds of TCMIs can be selected for the treatment of HCC, but studies on the direct comparison of the relative efficacy between two or more TCMIs are rare. Therefore, it is essential to conduct a network meta-analysis to compare the advantages and disadvantages of different TCMIs and analyse their respective characteristics to make the most suitable choice for patients in clinical practice. Network meta-analysis of TCMIs combined with TACE for the treatment of HCC is common, but a network meta-analysis of TCMIs combined with systemic chemotherapy for HCC has not yet been reported.

This study evaluated the efficacy of three TCMIs combined with systemic chemotherapy in the treatment of HCC, including Aidi injection, compound Kushen injection, and Kanglaite injection. The compositions of the three TCMIs are shown in Table 2.

Aidi injection is a compound traditional Chinese medicine preparation; that is, it is a combination of multiple traditional Chinese medicine extracts. The traditional Chinese medicines included in Aidi injection are Astragalus, ginseng, Acanthopanax senticosus, and cantharidin. *Astragalus* polysaccharides have hepatoprotective, antioxidative, and antitumour effects [34, 35]. Ginsenosides in various models of tumour cells and vascular endothelial cells show antitumour and antiangiogenic effects [36]. Acanthopanax senticosus has antitumour effects, which may be related to the inhibition of VEGF and VEGF mRNA expression [37]. Cantharidin also has an effect similar to that described above. In addition, it can increase white blood cells and reduce the occurrence of bone marrow suppression [38]. The theory of traditional Chinese medicine is that the combination of the four herbs can enhance their efficacy in HCC.

Compound Kushen injection, also known as Yanshu injection, is extracted from two kinds of traditional Chinese medicinal herbs: Sophora flavescens and Glabrous Greenbrier Rhizome. Experimental studies have confirmed that compound Kushen injection has an obvious killing effect on tumour cells, such as Hep and H22 cells, in vitro [39]. Moreover, research has shown that compound Kushen injection can significantly promote the expression of the tumour metastasis suppressor gene nm23 in BEL-7402 cells and inhibit the expression of CD44v6 in BEL-7402 cells [40]. Based on these data, compound Kushen injection may be selected as an additional treatment to inhibit the growth and metastasis of liver cancer cells.

Kanglaite injection is an extract from Coix seed, which is a traditional Chinese medicinal herb. The main active ingredient of Kanglaite injection is a triglyceride, which contains four types of fatty acids [41]. Kanglaite injection was first approved in 1997 in China for the treatment of HCC and approved by the US Food and Drug Administration (FDA) for a phase III clinical trial in 2015 [42]. A clinical study with 156 HCC patients comparing Kanglaite injection with systemic chemotherapy showed that Kanglaite injection had similar efficacy and safety when compared to chemotherapy. The chemotherapy regimen in the study was PAF (DDP + ADM+5-FU) [43]. The mechanisms of Kanglaite injection in the treatment of liver cancer are multifaceted. First, it can induce cancer cell apoptosis by activating proapoptotic factors such as p53, Fas, and caspase-3 [44, 45]. Second, it can inhibit the growth of HepG2 cells by stimulating anticancer immune function [46]. It can also induce apoptosis and cell cycle arrest through the PI3K/AKT pathway, thus enhancing the sensitivity of tumour cells to chemotherapy [47].

This study showed that the treatment efficacy was significantly enhanced by combining either Aidi injection, compound Kushen injection, or Kanglaite injection with chemotherapy. Although the efficacy of the three different kinds of injections was not significantly different, there seemed to be a tendency that Kanglaite injection was better than the other two in terms of ORR. This suggests that if further research with larger samples is carried out, significant differences may be achieved. These results should provide a reference for the clinical selection of TCMIs in adjuvant treatment assisting chemotherapy for HCC.

It is worth noting that combining TCMIs with chemotherapy could significantly increase the number of CD3^+^ T lymphocytes, CD4^+^ T lymphocytes, and the ratio of CD4^+^/CD8^+^ T lymphocytes. This showed that TCMIs could enhance the immune function of patients by increasing the activity of T lymphocyte subsets. Systemic chemotherapy often leads to a decline in patients' immune function. This is not only detrimental to tumour control but also brings a potential danger of virus reactivation to patients infected with hepatitis B [48, 49]. Therefore, patients with HCC could utilize such TCMIs to enhance immune function.

Some studies also reported OS and TTP. However, due to the small amount of data, a network meta-analysis was not carried out.

In terms of treatment safety, according to the results of this study, all kinds of treatment had tolerable adverse reactions, and the addition of TCMIs significantly reduced the incidence of adverse reactions. The safety of these three kinds of TCMIs was satisfactory, and TCMIs could further enhance the safety of chemotherapy and reduce patient suffering.

Our study showed that the TCMIs combined with systemic chemotherapy may be an effective and safe treatment option for patients with HCC. Furthermore, among the three TCMIs, Kanglaite injection has a tendency to outperform the other two injections in terms of ORR. This study laid the foundation for further research in the future. Moreover, our study focused on the role of TCMIs in combination with systemic chemotherapy rather than the interventional chemotherapy, which has not been reported before. Therefore, it is of considerable significance to carry out this study, which provides a basis for the application of systemic chemotherapy combined with TCMIs in patients with advanced HCC.

There are some aspects of this study that need to be improved; especially, the quality of the included literatures is not very satisfactory. Although all the literature included in this study were randomized controlled trials, only 5 of them (25%) described specific methods of random allocation. None of the included studies mentioned whether blindness and random allocation were used. Although ORR and CBR were unlikely to be influenced by lack of blindness, because they were based on image evaluations, KPS might be affected. This potential risk of bias may affect the authenticity and reliability of the results and lead to the reduced power of the test.

In future RCTs of TCMIs, clinical researchers should reduce the bias in the trial process as much as possible to improve the quality of the research evaluating the efficacy of TCMIs in the treatment of HCC. If possible, an RCT directly comparing different kinds of TCMIs should be conducted to evaluate their efficacy.

## Figures and Tables

**Table 1:** Details of the characteristics of the studies.

Study ID (author, year)		N (E/C)	M/F	Average age (E/C)	Stage	KPS (E/C)	Treatment (E/C)	Duration	Outcomes^c^
Study 1 (Huang et al., 2002) [10]		30/28	43/15	57.6/56.2	II–IV	NR	Aidi Inj+5Fu/5Fu	28d^∗^4	1, 2, 3, 4, 5
Study 2 (Hou et al., 2017) [11]		43/43	45/41	55.7/58.2	II–IV	69.27/71.34	Aidi Inj+5Fu/5Fu	30d^∗^1	1, 2, 3, 4
Study 3 (Hou, 2018) [12]		47/47	57/37	54.57/56.24	II–IV	62.23/62.41	Aidi Inj+CAFI/CAFI	28d^∗^2	1, 2
Study 4 (Hongwen et al., 2012) [13]		36/36	48/24	52/54	NR	≥60/≥60	Aidi Inj+FOLFOX4/FOLFOX4	28d^∗^4	1, 2, 3, 4, 6
Study 5 (Zhang, 2018) [14]		35/35	33/37	54.93/55.21	NR	62.19/62.34	Aidi Inj+XELOX/XELOX	14d^∗^4	1, 2
Study 6 (Zhang, 2018) [15]		35/34	39/30	58.60/58.20	III–IV	NR	Aidi Inj+XELOX/XELOX	21d^∗^4	1, 2, 4
Study 7 (Zhang and Wang, 2007) [16]		24/18	30/12	Range: 32–75^a^	III–IV	≥60/≥60	Aidi Inj+DDP+5Fu/DDP+5Fu	28d^∗^2	1, 2, 3, 4
Study 8 (Shi and Wang, 2011) [17]		35/35	36/34	51.3/52.9	III–IV	NR	Aidi Inj+DDP+EPI/DDP+EPI	21d^∗^2	1, 2, 3, 5
Study 9 (Liu et al., 2013) [18]		30/30	33/27	55/56	II–III	NR	Kushen Inj+MAF/MAF	21d^∗^3	1, 2, 4
Study 10 (Chen and Xun, 2006) [19]		16/14	19/11	63.4/65.3	II–III	≥50/≥50	Kushen Inj+FAP/FAP	28d^∗^2	1, 2, 3, 4
Study 11 (Jin, 2016) [20]		36/36	42/30	54.3^a^	II–IV	NR	Kushen Inj+GEMOX/GEMOX	21d^∗^2	1, 2, 4
Study 12 (He, 2018) [21]		43/43	49/37	60.4/59.2	II–IV	64.76/63.98	Kushen Inj+FOLFOX6/FOLFOX6	21d^∗^6	1, 2, 4
Study 13 (Li et al., 2018) [22]		40/40	51/29	53.8/51.3	III–IV	NR	Kushen Inj+MAF/MAF	21d^∗^3	1, 2
Study 14 (Shi, 2011) [23]		30/30	35/25	52.8/53.4	IIIb–IV	≥60/≥60	Kushen Inj+GP/GP	30d^∗^2	1, 2, 4
Study 15 (Wu et al., 2002) [24]		30/30	53/7	42/43	II–III	NR	Kushen Inj+FUDR/FUDR	20d^∗^1	1, 2, 3, 4
Study 16 (Guan et al., 2006) [25]		20/18	NR	Range: 26–65^a^	III	≥50/≥50	Kushen Inj+GEMOX^b^/GEMOX	15d^∗^4	1, 2, 3, 5
Study 17 (Yao, 2015) [26]		30/30	35/25	55/56	NR	NR	Kushen Inj+GEMOX/GEMOX	21d^∗^2	1, 2
Study 18 (Xu et al., 2010) [27]		38/37	58/17	48.5^a^	II–III	NR	Kanglaite Inj+CAP/CAP	21d^∗^2	1, 2, 3, 4, 6, 7
Study 19 (Ma et al., 2017) [28]		43/43	38/48	54.8/53.7	NR	56.24/58.49	Kanglaite Inj+FOLFOX6/FOLFOX6	21d^∗^2	1, 2, 8
Study 20 (Li et al., 2014) [29]	75/75	79/71	53.2/52.1	I–IV	NR	Kanglaite Inj+ADM/ADM	30d^∗^2	1, 2, 3, 4, 5, 8

N: number; E: experimental group; C: control group; M: male; F: female; NR: not reported; Inj: injection. ^a^Merged data of two groups; ^b^there were 3 kinds of chemotherapy protocol in this article: GEMOX, GEM+5–Fu+THP, and HCPT+FT207+THP; ^c^1=objective response rate; 2=clinical benefit rate; 3=KPS; 4=adverse reactions; 5=overall survival; 6=time to progress; 7=median survival time; 8=immune function.

**Table 2:** Compositions of the three TCMIs.

TCMIs	Compositions
Aidi injection	*Mylabris phalerata* Pallas
	*Panax ginseng* C. A. Mey
	*Astragalus propinquus* Schischkin
	*Eleutherococcus senticosus* (Rupr. and Maxim.) Maxim

Compound Kushen injection	*Sophora flavescens* Aiton
	*Heterosmilax japonica* Kunth

Kanglaite injection	*Coix lacryma-jobi L*.

## Data Availability

All data generated and used during the study are available from the corresponding author on request.
